# Implementing resilience engineering for healthcare quality improvement using the CARE model: a feasibility study protocol

**DOI:** 10.1186/s40814-016-0103-x

**Published:** 2016-10-12

**Authors:** J. E. Anderson, A. J. Ross, J. Back, M. Duncan, P. Snell, K. Walsh, P. Jaye

**Affiliations:** 1 Florence Nightingale Faculty of Nursing and Midwifery, King’s College London, James Clerk Maxwell Building, 57 Waterloo Road, London, SE1 8WA UK; 2 Dental School, School of Medicine, University of Glasgow, Glasgow, UK; 3 Guy’s and St. Thomas’ NHS Foundation Trust, London, UK; 4 BMJ Learning, BMJ, London, UK; 5 Simulation and Interactive Learning (SaIL) Centre, St Thomas’ Hospital, King’s Health Partners, London, UK

**Keywords:** Patient safety, Resilient healthcare, Quality improvement, Resilience engineering, Mixed methods, Organisational resilience, Quality of care

## Abstract

**Background:**

Resilience engineering (RE) is an emerging perspective on safety in complex adaptive systems that emphasises how outcomes emerge from the complexity of the clinical environment. Complexity creates the need for flexible adaptation to achieve outcomes. RE focuses on understanding the nature of adaptations, learning from success and increasing adaptive capacity. Although the philosophy is clear, progress in applying the ideas to quality improvement has been slow. The aim of this study is to test the feasibility of translating RE concepts into practical methods to improve quality by designing, implementing and evaluating interventions based on RE theory. The CARE model operationalises the key concepts and their relationships to guide the empirical investigation.

**Methods:**

The settings are the Emergency Department and the Older Person’s Unit in a large London teaching hospital. Phases 1 and 2 of our work, leading to the development of interventions to improve the quality of care, are described in this paper. Ethical approval has been granted for these phases. Phase 1 will use ethnographic methods, including observation of work practices and interviews with staff, to understand adaptations and outcomes. The findings will be used to collaboratively design, with clinical staff in interactive design workshops, interventions to improve the quality of care. The evaluation phase will be designed and submitted for ethical approval when the outcomes of phases 1 and 2 are known.

**Discussion:**

Study outcomes will be knowledge about the feasibility of applying RE to improve quality, the development of RE theory and a validated model of resilience in clinical work which can be used to guide other applications. Tools, methods and practical guidance for practitioners will also be produced, as well as specific knowledge of the potential effectiveness of the implemented interventions in emergency and older people’s care. Further studies to test the application of RE at a larger scale will be required, including studies of other healthcare settings, organisational contexts and different interventions.

## Background

Despite focused worldwide efforts over a decade and a half to improve the quality of healthcare, progress remains frustratingly slow [1, 2]. Adverse events result in death or harm to many patients and require financial and other resources to be devoted to ameliorating harm and compensating the victims of patient safety incidents [3]. Preventing problems such as wrong site surgery and ensuring the delivery of evidence-based treatments appear to have evidence-based solutions, but in practice these are either not implemented or not effective. The persistence of care quality problems implies that further progress will only be possible if we find more effective quality improvement strategies.

Resilience engineering (RE) is an emerging paradigm for understanding and improving complex adaptive systems such as healthcare [4–6]. In the last 10 years, a multi-disciplinary community of researchers has enthusiastically debated and documented the philosophical basis of RE. A series of foundational monographs has been published from which the core concepts can be identified and the development of the key ideas can be traced [7–12]. These concepts are further explained in the rest of this section.

RE has evolved by drawing major influences from diverse disciplines including cognitive psychology, safety science, engineering and human factors. A major impetus for its development was dissatisfaction with prevailing approaches to safety improvement which are thought to have had limited success because they emphasise retrospective incident analysis, reactive measures targeted at past problems, the ubiquity of human error as an explanation and the control of work through procedural compliance and monitoring of outcomes [13, 14]. Although RE and positive deviance [15] share similar concerns, such as a desire to move away from retrospectivity and learn from what goes right, RE is a coherent conceptually well-developed field in its own right.

Despite enthusiastic support from practitioners and safety professionals in fields as diverse as nuclear power [16, 17], petroleum [18], process industries [19], aviation [20, 21], rail transport [22] and healthcare [23–25], the development of an evidence base showing the benefits of resilience principles for improving the quality of care has been slow [26]. This can partly be attributed to the difficulty of operationalising the concepts in order to investigate them empirically. A clear definition of the variables involved and how they will be assessed, measured or described is necessary for effective research, and this has proved challenging. In this study, we are investigating the feasibility of applying a resilience approach to improve the quality and safety of care using a model of resilience to operationalise the key concepts. The Concepts for Applying Resilience Engineering (CARE) model, explicated in later sections, underpins this study and will be used to guide data collection, analysis and interpretation.

RE is at an early developmental stage. Nevertheless, it is possible to identify the main concepts. Resilience can be defined as ‘the ability of the health care system (a clinic, a ward, a hospital, a county) to adjust its functioning prior to, during, or following events (changes, disturbances, and opportunities), and thereby sustain required operations under both expected and unexpected conditions.’ [27]. Several important implications follow from this. First, RE focuses on the organisational level (unlike the traditional focus on individual behaviour) and the importance of adjustment to varying conditions in the work environment rather than rigid compliance with standards. RE is not concerned with individual psychological resilience or coping, but with organisational processes that enable a team or unit to adapt successfully. The focus on adjustment is linked to the recognition that complex systems such as healthcare are intractable [28, 29]; work cannot be fully specified in advance, and so the delivery of successful outcomes relies on the ability of workers and teams to adjust to fluid situations and thereby to actively create safe outcomes.

Second, because RE proposes that variability in the environment creates the need for adjustment, acceptable and unacceptable outcomes are thought to emerge from the same adaptive processes as workers adjust flexibly [30] to cope with such problems as unexpectedly high patient numbers, lack of equipment or staff shortages. As well as responding to variable conditions, workers have a proactive tendency to adapt creatively as they take control of the working environment and strive to increase efficiency and reduce workload [31]. In this paradigm, adverse events are theorised to occur because the ability to adjust appropriately has been overwhelmed by environmental conditions. Workers are therefore seen as a key strength of the system in contrast to other improvement approaches which emphasise their vulnerability to errors and mistakes and cast them as the weak link in the system [32, 33].

**Fig. 1:**
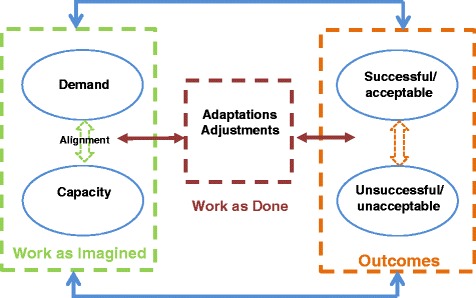
Concepts for Applying Resilience Engineering (CARE) model

Third, the need for adjustment and the ubiquity of adaptation means that there is a difference between work as imagined (WAI) in protocols, procedures and targets and work as done (WAD) in practice [34–37]. The gap between WAI and WAD has been a focus of a number of RE studies, and the need to understand adaptations and how work is accomplished has led to a focus on understanding and describing everyday clinical work [38, 39]. The gap between WAI and WAD is viewed as a danger to safety since real working processes remain undescribed and poorly understood. One assumption is that the gap between WAI and WAD can and should be narrowed or closed completely. Our view (see Fig. 1) is that human agency will always lead to innovations, shortcuts, workarounds and adaptations, and some misalignment between protocol and everyday work will always exist, creating an ever present need for adjustment to suit local context.

Finally, a further insight is that if successful and unsuccessful outcomes arise from adaptive processes, it makes sense to study what goes right rather than focus entirely on what goes wrong, as exemplified by current safety practices such as incident reporting, run charting of events and root cause analysis [40]. Studying what goes right makes sense because there is a much larger sample of successful than unsuccessful events [40] and it potentially sheds light on otherwise unrecognised and unspecified pathways to success which are key to resilient organisations.

The rationale for these key concepts (complexity causing variability, the need for flexible working, the distinction between WAI and WAD and the importance of learning from what goes right) is clear and based on empirical evidence and philosophical reasoning. These principles seem particularly appropriate to healthcare given the well-documented variability of processes and the complexity of the environment [41, 42]. The next step is to answer a number of important questions including the following:

- How does studying adaptability and adjustments in situ help us to improve quality?
- Does improvement rest on aligning WAI and WAD more closely or improving adaptive capacity?
- How can adaptive capacity be increased and will this lead to improvements in the quality of care?
- If we are to study ‘what goes right’, how do we select the correct unit of analysis and what to study, given that a multitude of things ‘go right’ cognitively, socially, organisationally?

This protocol outlines a study to test the feasibility of translating emerging theory and practice in RE into practical steps to improve the quality and safety of care by researching, developing and implementing interventions based on RE theory and evaluating them in terms of quality, safety, costs and benefits. The study is concerned with whether RE can be practically implemented in two clinical settings and with providing intermediate evidence of its efficacy prior to potential full-scale testing [43].

### The CARE model—operationalising resilience concepts

The study is based on a theoretical model of the key resilience concepts and the relationships between them. The Concepts for Applying Resilience Engineering (CARE) model provides a framework for studying how organisational resilience is manifest in healthcare, how it contributes to outcomes and how it might be strengthened. The model contains various implicit hypotheses about resilience mechanisms; it is thus likely to be developed more fully as the empirical work progresses. The model, shown in Fig. 1, is an abstraction of important concepts and is not intended to reflect the full complexity of clinical work but to focus attention on important theoretical constructs to be investigated empirically.

### Work as imagined and work as done

In the CARE model, WAI is conceptualised as an intended, or imagined, alignment between demands in the system (such as patient numbers, acuity, quality standards) and capacity to meet those demands. Organisations plan staffing levels, purchase equipment, train staff and devise procedures and protocols to meet the demand as it is imagined based on past experience and future projections. Demand and capacity can, however, never be completely aligned because of the complex nature of the system; there will always be unforeseen demands, variance and interactions that require workers to adjust in situ. In addition, people are not passive; they do not simply comply with protocol but naturally adapt and innovate as part of taking control over their environments. Thus adaptation per se and specific adjustments to variability are an inevitable consequence of the healthcare environment. In the CARE model, WAD refers to the adjustments that are required to accommodate these misalignments and the natural variability in how tasks are carried out. As postulated in RE, predicting acceptable and unacceptable outcomes depends to a large extent on understanding these crucial dynamics of WAD in different demand-capacity circumstances.

### Outcomes

Outcomes are viewed broadly and include consequences for patients, staff and the organisation. Success and failure are not fixed categories but subject to interpretation and judgement based on the context. Competing demands and a complex network of stakeholders with diverse needs mean that goals are prioritised and often traded off to produce acceptable outcomes [38, 44] given contextual factors such as patient characteristics, available staff expertise and time, and patient load. For example, it might not be possible to meet standards for the optimal patient experience when there is an influx of high-acuity patients who have higher than anticipated care needs. Meeting targets for patient flow in the emergency department might not always result in high-quality patient care, yet this is a required government performance target. Staff in these situations will prioritise (hence trade off) conflicting goals to achieve the best outcomes. Divergent and variable patient, NHS, organisational and staff perspectives will always partly determine which goals are prioritised [45].

### Systems perspective

RE is based on insights from systems theory, and accordingly the CARE model contains feedback loops and recursive structures. Specifically, there is a non-linear structure, whereby, for example:

- Outcomes are not end points. They can affect all other aspects of the model. For example, consistently successful outcomes might reduce capacity if there is a perceived need for fewer staff.
- Adaptations or adjustments that are deemed successful may be codified in procedures and protocols and become incorporated into WAI; they will then be subject to adaptations and adjustments themselves as the system state changes. Many local protocols such as escalation policies, for example, contain elements that were once informal local adjustments to misalignment or variability.
- No particular adjustment or adaptation is ‘right’ or ‘wrong’ in itself. In a slightly different context, it may prove maladaptive due to the complex interactions involved.
- Elements of the healthcare system are not fixed in terms of whether they provide capacity or introduce demand. For example, a training course can, from one perspective, be seen as a demand requiring the release of staff, but might simultaneously be seen as increasing capacity in terms of new skills or knowledge.

### Why this study is needed

Problems with the quality of healthcare persist despite efforts to prevent harm to patients [2], suggesting that there is a need for new approaches to quality improvement. Although the theoretical principles of RE are now well established, most studies from this perspective have focused on describing specific clinical processes and adjustments to understand work as it is done [26]. None to our knowledge has investigated organisational resilience in several departments, used the data to design interventions and implemented interventions to increase resilience and improve the quality of care. Testing the feasibility of applying these concepts in practice to improve the quality and safety of care is the next vital stage.

### Aims

The overall aim of this study is to develop and test interventions based on RE principles to improve the quality of care. In order to achieve this, the study will develop methods and tools for investigating quality and safety from a resilience perspective. The study is formative in that interventions will be designed following in-depth research to understand the pressures and problems that threaten quality and current levels of resilience. The rationale for a formative approach is that interventions need to be designed based on an accurate understanding of current processes and how outcomes are achieved (WAD). Interventions and therefore methods cannot be fully specified in advance. The objectives are to:

1. Understand and map resilience in two clinical units, including how WAI differs from WAD, how adjustments are created and how outcomes emerge from the interplay of misalignments and adjustments
2. Building on these insights, design interventions in collaboration with clinical teams to increase resilience and improve quality
3. Implement interventions
4. Evaluate interventions in terms of quality, safety, costs and benefits

The study will produce evidence about the feasibility of implementing an RE approach to quality improvement, as well as evidence about its potential effectiveness. Further theoretical development of RE principles based on experience operationalising the approach in clinical settings will also be generated.

### Study design

This is a mixed methods study with three phases: ethnography, intervention design and implementation, and evaluation. The focus of ethnographic data collection and analysis will be on understanding the links between demand and capacity misalignments, adjustments, adaptations and outcomes to understand at a deep level the context of clinical work and the situated actions of clinical staff as they grapple with the variability and complexities of the environment (see Fig. 1).

Intervention design and implementation will be collaborative, involving clinical staff in the interpretation of the ethnographic data and the design of feasible interventions. This will be achieved through a series of interactive workshops involving staff from all professional groups and levels. Interventions will be evaluated in terms of changes in quality and safety metrics and the impact on clinical processes. In this paper, phases 1 and 2 will be described, the outputs of which will be the designed interventions. Evaluation design is dependent on the outcomes of the earlier phases and therefore cannot be specified in advance.

### Settings

The study will be carried out in two departments at a large London teaching hospital: Accident and Emergency (A and E) and the Older Person’s Unit. In both specialities, there are well-documented quality problems. Timely flow through A and E is a proxy measure of quality as increased time in the department and crowding have both been associated with poorer outcomes [46, 47]. Despite the imposition of targets mandating that 90 % of patients should be seen and discharged within 4 h, target breaches remain common in the NHS [48]. Other common quality problems include wrong diagnoses and miscommunication [49]. Older people are the greatest users of health-related services [50, 51]. Concerns about safety, dignity, compassion and high-quality care have been highlighted [52, 53], and the quality of care received by older people is high on the policy agenda. For example, the poor quality of older people’s care has been recently highlighted by the care failings at the Mid Staffordshire Trust [54].

In addition to the potential to improve care quality, these units were chosen because the nature of the clinical work varies, allowing examination of how different demands and levels of predictability and controllability affect resilience. Table 1 summarises the differences between the two units.

**Table 1:** Comparison of demands in emergency care and care of older people

Demands	Older Person’s Unit	Accident and Emergency
Clinical problems	Multiple chronic problems Sensory and cognitive deficits common Patient numbers and demographics relatively stable	Acute problems Patient numbers vary greatly Variable patient demographics
Predictability	Some ability to predict demands due to stable patient numbers Patient acuity levels unpredictable both for individuals and wards	Ability to predict busy times of the day and week Low ability to predict specific demands at any one time, especially patient numbers and acuity
Multi-disciplinary team (MDT)	Co-located MDT Dispersed community-based MDT	MDT dispersed
Timescales	Co-ordination of care over time required	Co-ordination required over short timescales

The success of the study will depend on close engagement with clinical teams, and for this reason, the research team includes a senior clinician from each area who will assist by raising awareness of the study, facilitating access, interpreting data and contributing to the progress of the study.

## Methods

Although the study is based on RE theory and therefore cannot be described as a classic study in the grounded theory tradition, it will adopt grounded theory concepts such as a formative research design, iterative cycles of data collection and analysis, and a focus on theory development [55]. There are three distinct but related phases: ethnography, intervention development and evaluation. The multi-phase design of the study mirrors the stages of the MRC complex intervention framework for complex interventions which emphasises the importance of an intervention development phase followed by testing and evaluation [56]. Since the interventions will not be specified until the end of phase 2, this protocol will focus on phases 1 and 2 only.

### Phase 1: ethnography of resilient performance

Phase 1 will involve in-depth ethnographic fieldwork in both clinical settings to understand:

1. What misalignments between demand and capacity occur and why?
2. What pressures, problems or goals are clinicians responding to when they create new ways to achieve outcomes?
3. How and under what circumstances do adjustments or adaptations lead to successful and unsuccessful outcomes?

#### Procedures

Data will be collected using non-participant observations of clinical work, including but not limited to shadowing of individual staff members, semi-structured interviews with staff (*n* = 15 in each unit) and analysis of documents such as meeting minutes, reports and policies. Data will be collected concurrently in each setting, and researchers will work across both settings so that comparisons between the settings can inform ongoing data collection strategies and interpretation.

Observations will be conducted in stages. The first exploratory sweep will be focused on the whole clinical environment and will identify staff roles and responsibilities, processes and procedures, flows of information and communication, co-ordination mechanisms, and supporting tools and technology. A key aim will be to identify areas of interest for more focused and targeted observations in subsequent sweeps, which will involve in-depth observations of important mechanisms such as handover, multi-disciplinary meetings and ward rounds. These will be observed by shadowing clinical staff and may involve short discussions and facilitated reflection from staff on aspects of the CARE model. Full descriptive field notes will be produced immediately after each observational session. Participant bias caused by sensitivity to the presence of the researcher is a recognised risk in qualitative observational studies [57] but can be minimised by ensuring that observers have frequent presence in the research setting, which can lead to habituation to their presence, researcher’s sensitivity to and respect for clinicians’ concerns, and their ability to build relationships and trust. The researchers in this study are experienced in healthcare research, and the research team, including clinicians, will be available to discuss and resolve any emerging difficulties. The longitudinal nature of the study will assist with reducing participant bias.

Semi-structured interviews with key informants will be conducted to explore and follow up issues identified in observations and to gain insight into processes or areas for further observation. All staff in all professional groups and all levels will be invited to participate in interviews following the provision of written information about the study. Staff members who were observed will first be invited to interview to allow further clarification or exploration of practice. Questions will be open ended and exploratory to focus on how clinical work is accomplished, variability, adaptations, problems and challenges. Interviews will be audio recorded and fully transcribed for analysis with participants’ consent.

Given the qualitative, interpretive nature of ethnographic studies, researcher bias is a risk. In line with the aims of the study, researchers will be using a resilience lens to observe practice. The risk of bias will be reduced by having two researchers sharing the work, ensuring that differences in interpretation can be surfaced, discussed and resolved. In addition, the whole research team will meet regularly to discuss interpretations and perspectives on the data.

#### Data analysis

Analysis will proceed in two stages. First, a combined deductive-inductive approach will be used to thematically analyse the data. A coding scheme will be developed based on the elements of the CARE model and important themes in the data not captured by the model. Categories will be developed by constantly refining the coding scheme, and comparisons between different respondents, different clinical units and different processes will be made.

Second, data from different sources will be synthesised to create resilience narratives describing trajectories of activity linking misalignments of demand and capacity, adjustments, adaptations and outcomes. Specifically, the narratives will focus on how adjustments and adaptations (WAD) mediate between pressures caused by misalignments of demand and capacity, and outcomes. Resilience narratives will thus be context rich and context dependent and will be used in an exploratory way to inform the intervention development. The aim is not to describe linear cause and effect relationships but to use narratives to uncover hypothesised relationships between variables that can be tested in the subsequent quality improvement interventions. The outcomes from phase 1 will be an in-depth description of resilient performance in each setting and identified opportunities for strengthening resilience through interventions.

### Phase 2: collaborative design and implementation of interventions

Phase 2 will involve collaborating with clinicians to design and implement interventions based on the insights generated in phase 1. An inclusive collaborative process will be used involving a multi-disciplinary advisory group in each setting in a series of workshops. This will increase the relevance and feasibility of the interventions and increase the likelihood of successful implementation. All staff on the units will be invited to participate with the aim of including all staff groups and levels. The aim will be to clearly describe the intervention, its causal mechanisms, expected effects and a clear process for its implementation. Workshops will initially involve the presentation and discussion of emerging findings from phase 1. As the study progresses, the focus will change to possible interventions, design of a change process, and discussion of the process of implementation and the effects.

#### Procedures

A clinical advisory group with representatives from all disciplines and a range of staffing grades will be established in each unit. Workshops to discuss and reflect on the results of phase 1 will be held as these results emerge. The group will advise on emerging results, assist with focusing the ongoing data collection and discuss potential interventions. Membership of the advisory groups may change due to staff turnover, shift work and clinical demands. The output of this phase will be agreement with clinical leaders and teams and detailed proposals for the interventions to be implemented.

### Ethical approval

Ethical approval has been sought and granted by the Psychiatry, Nursing and Midwifery Research Ethics Subcommittee (PNM RESC) of King’s College London (PNM 14/15-17) to conduct phases 1 and 2 of the study. The design of the interventions will be based on the outcomes of phases 1 and 2, and so they cannot be specified at this time. Further applications for ethical approval will be submitted when the interventions have been designed.

### Patient and public involvement

The development of interventions in phase 2 will involve patients and the public in the design of the interventions if this is appropriate. Some interventions will likely focus on the organisation of work and how to strengthen processes to deliver outcomes. In these cases, the focus of the intervention will be one step removed from the delivery of hands-on care. For example, co-ordination between team members is important for resilience, but an intervention to increase team co-ordination is not necessarily an issue that best benefits from patient and public involvement as it does not aim to change the delivery of care and is not patient facing. In contrast, for interventions that focus on direct interactions with patients, families and relatives, it would be vital to involve patients and the public to understand what is important to them and how they would like the service to address their needs. In phase 2, we will work with our clinical partners to involve patients and the public in the design of the interventions as appropriate, drawing on the networks of contacts in the hospital. Both study units have existing processes for involving patients and the public in designing and reflecting upon care, and these views will be elicited in the intervention design workshops.

### Study status

The study is ongoing. Data collection commenced in October 2014 and is continuing. Intervention development will be completed by the end of 2015, and implementation and evaluation will commence in early 2016 alongside evaluation.

## Discussion

This study will be the first to our knowledge to operationalise RE principles within a quality improvement initiative and produce a validated model of resilience in clinical work. It is therefore important for the development of RE theory and practice and will add new knowledge about the feasibility of applying RE to improve healthcare quality. The CARE model will be tested by its application in practice and may be elaborated or revised based on the findings. This will potentially add value to quality improvement knowledge especially given recent criticisms of the lack of a systems approach in patient safety research [58] and the slow progress of quality improvement in general [1].

In our experience, clinicians find the philosophy and principles of RE attractive because it accurately portrays the constant variability of clinical work, the need for adjustment and the important role of flexible adaptation in producing outcomes. There is demand from practitioners for guidance to apply RE principles, and there is now a clear need to test the ideas in practice.

Outputs from this study may include tools, methods and guidance on implementation processes. If appropriate, findings, tools and methods will be disseminated directly to the health service via a handbook designed to assist the implementation of RE principles in practice. Workshops and symposia for clinical practitioners may also be used to transfer skills. The findings will directly inform future approaches to RE and quality improvement generally.

### Strengths and limitations

The key strengths of this study are the use of mixed methods to study resilience from multiple perspectives and the strong theoretical basis for the approach. Mixed methods allow triangulation of results, highlight agreements and conflicts between data from different sources, and ensure a complete and nuanced understanding of complex phenomena [59]. The focus on operationalising RE theory is timely since care quality problems have been difficult to solve and the limitations of traditional approaches have been highlighted. The study will add significantly to the evidence base on RE in healthcare and whether and how it can contribute to improving quality.

There are, however, several limitations to the study. First, the study is based in a single NHS Trust with no control. This limits our capacity to account for the influence of trust-level factors on resilience. However, the purpose of this study is to operationalise and test RE theory in quality improvement, and so we will need to study resilience in enough depth in one organisation to test the feasibility of the approach. In-depth and nuanced understanding is a characteristic of ethnographic research and is a major advantage for uncovering the realities and subtleties of clinical work that are normally hidden [60].

Second, although generalising the results to other organisations may not be possible, the aim is to understand in depth the processes in one organisation before conducting future studies in which comparisons are made between organisations. The study will contrast two units in the same organisation, allowing some ability to understand organisational factors which will be common to both units. Data collection inevitably will involve interpretive processes, but we will increase reliability by having two researchers sharing insights and regular team meetings in which interpretations are shared and discussed. Moreover, the research team is multi-disciplinary, including members with diverse clinical and academic backgrounds and therefore ensuring different perspectives on the data are explored.

Third, clinical staff will be involved in the design of interventions, but it is possible that the advisory groups may not be representative and therefore the interventions may not be accepted within the unit or directly transferrable to others. Finally, the clinical environment is constantly experiencing turnover of staff, procedures, policies, teams and equipment. In such an environment, there are many factors that may influence behaviour, and it may not be feasible to link interventions with observed changes. Future studies will be needed to investigate the interaction of varying contextual factors (organisational, specialty, unit) to produce resilience and suggest different ways in which it can be strengthened.

## Abbreviations

CARE: Concepts for Applying Resilience Engineering
RE: Resilience engineering
WAD: Work as done
WAI: Work as imagined

## References

[1] Vincent C, Aylin P, Franklin BD, Holmes A, Iskander S, Jacklin A, Moorthy K (2008). Is health care getting safer?. BMJ.

[2] Vincent C, Burnett S, Carthey J (2014). Safety measurement and monitoring in healthcare: a framework to guide clinical teams and healthcare organisations in maintaining safety. BMJ Qual Saf.

[3] Dept. Health (2000). An organisation with a memory.

[4] Braithwaite J, Clay-Williams R, Nugus P, Plumb J, Hollnagel E, Braithwaite J, Wears R (2013). Health care as a complex adaptive system. Resilient health care.

[5] Robson R, Hollnagel E, Braithwaite J, Wears R (2013). Resilient health care. Resilient health care.

[6] Robson R, Wears R, Hollnagel E, Braithwaite J (2015). ECW in complex adaptive systems. Resilient health care volume 2. The resilience of everyday clinical work.

[7] Hollnagel E, Woods DD, Leveson N (2006). Resilience engineering: concepts and precepts.

[8] Hollnagel E, Nemeth CP, Dekker S (2008). Resilience engineering perspectives volume 1: remaining sensitive to the possibility of failure.

[9] Nemeth C, Hollnagel E, Dekker S (2009). Resilience engineering perspectives volume 2: preparation and restoration.

[10] Hollnagel E, Paries J, Woods D, Wreathall J (2011). Resilience engineering in practice: a guidebook.

[11] Hollnagel E, Braithwaite J, Wears RL (2013). Resilient health care.

[12] Wears RL, Hollnagel E, Braithwaite J. editors. Resilient Health Care Volume 2: The Resilience of Everyday Clinical Work. Farnham, UK: Ashgate Publishing; 2015.

[13] Hollnagel E, Braithwaite J, Wears R, Hollnagel E, Braithwaite J, Wears RL (2013). Preface: on the need for resilience in health care. Resilient health care.

[14] Hollnagel E (2013). A tale of two safeties. Int Electron J Nucl Saf Simul.

[15] Lawton R, Taylor N, Clay-Williams R, Braithwaite J. Positive deviance: a different approach to achieving patient safety. BMJ Qual Saf. 2014;23:880–883. doi:10.1136/bmjqs-2014-003115. 10.1136/bmjqs-2014-003115 PMC4215344 25049424

[16] Furniss D, Back J, Blandford A, Hildebrandt M, Broberg H (2011). A resilience markers framework for small teams. Reliab Eng Syst Safe.

[17] Kitamura M, Nemeth C, Hollnagel E (2014). Resilience engineering for safety of nuclear power plant with accountability. Resilience engineering in practice, volume 2: becoming resilient.

[18] Hollnagel E, Tveiten C, Albrechtsen E. Resilience engineering and integrated operations in the petroleum industry. Report published by Norwegian University of Science and Technology Trondheim; 2010.

[19] Dinh LT, Pasman H, Gao X, Mannan MS (2012). Resilience engineering of industrial processes: principles and contributing factors. J Loss Prevent Proc.

[20] Da Mata TF, Gajewski DW, Hall CK, Lacerda MC, Santos AG, Gomes JO, Woods DD. Application of resilience engineering on safety in offshore helicopter transportation. Systems and Information Engineering Design Symposium IEEE; 2006. p. 228–33.

[21] Cabon P, Deharvengt S, Berechet I, Grau J, Maille N, Mollard R, Hollnagel E, Paries J, Woods DD, Wreathall J (2011). From flight time limitations to fatigue risk management systems—a way toward resilience. Resilience engineering in practice: a guidebook.

[22] Hale A, Heijer T, Hollnagel E, Woods DD, Leveson N (2006). Is resilience really necessary? The case of railways. Resilience engineering: concepts and precepts.

[23] Patterson ES, Woods DD, Cook RI, Render M (2007). Collaborative cross-checking to enhance resilience. Cogn Technol Work.

[24] Jeffcott S, Ibrahim J, Cameron P (2009). Resilience in healthcare and clinical handover. Qual Saf Health Care.

[25] Miller A, Xiao Y (2007). Multi-level strategies to achieve resilience for an organisation operating at capacity: a case study at a trauma centre. Cogn Technol Work.

[26] Anderson JE, Ross A, Jaye P (2013). Resilience engineering in healthcare: moving from epistemology to theory and practice. In proceedings of the fifth resilience engineering symposium.

[27] Wears RL, Hollnagel E, Braithwaite J. Preface. In Wears RL, Hollnagel E, Braithwaite, J, editors. Resilient health care, Volume 2: The resilience of everyday clinical work. Farnham, UK: Ashgate; 2015. p xxvii.

[28] Hollnagel E (2012). Coping with complexity: past, present and future. Cogn Technol Work.

[29] Hollnagel E (2012). FRAM: the functional resonance analysis method: modelling complex socio-technical systems.

[30] Braithwaite J, Wears R, Hollnagel E (2015). Resilient health care: turning patient safety on its head. Int J Qual Health Care.

[31] Cook R, Rasmussen J (2005). “Going solid”: a model of system dynamics and consequences for patient safety. Qual Saf Health Care.

[32] Nemeth C, Nunnally M, O’Connor M, Cook R. Creating resilient IT: how the sign-out sheet shows clinicians make healthcare work. In American Medical Informatics Association Annual Symposium Proceedings; 2006*.* p*.* 584. PMC1839397 17238408

[33] Sujan M, Spurgeon P, Cooke M, Wears RL, Hollnagel E, Braithwaite J (2015). Translating tensions into safe practices through dynamic trade-offs: the secret second handover. Resilient health care volume 2: the resilience of everyday clinical work.

[34] Patterson ES, Rogers ML, Chapman RJ, Render ML (2006). Compliance with intended use of bar code medication administration in acute and long-term care: an observational study. Hum Factors.

[35] Dekker S (2003). Failure to adapt or adaptations that fail: contrasting models on procedures and safety. Appl Ergon.

[36] Debono D, Braithwaite J, Wears RL, Hollnagel E, Braithwaite J (2015). Workarounds in nursing practice in acute care: a case of a Health Care Arms Race?. Resilient health care volume 2: the resilience of everyday clinical work.

[37] Nemeth CP, Cook RI, Wears RL (2007). Studying the technical work of emergency care. Ann Emerg Med.

[38] Wears R, Schubert C, Hunte G, Wears RL, Hollnagel E, Braithwaite J (2015). Individual-collective trade-offs: implications for resilience. Resilient health care volume 2: the resilience of everyday clinical work.

[39] Ross AJ, Anderson JE, Kodate N, Thompson K, Cox A, Malik R (2014). Inpatient diabetes care: complexity, resilience and quality of care. Cogn Technol Work.

[40] Hollnagel E (2014). Safety-I and safety-II: the past and future of safety management.

[41] Clay-Williams R, Hounsgaard J, Hollnagel E (2015). Where the rubber meets the road: using FRAM to align work-as-imagined with work-as-done when implementing clinical guidelines. Implement Sci.

[42] Waring J, McDonald R, Harrison S (2006). Safety and complexity: inter-departmental relationships as a threat to patient safety in the operating department. J Health Organ Manage.

[43] Bowen D, Kreuter M, Spring B, Cofta-Woerpel L, Linnan L, Weiner D, Bakken S, Kaplan C, Squiers L, Fabrizio C, Fernandez M (2009). How we design feasibility studies. Am J Prev Med.

[44] Hoffman RR, Woods DD (2011). Beyond Simon’s slice: five fundamental trade-offs that bound the performance of macrocognitive work systems. IEEE Intell Syst.

[45] Woods D, Hollnagel E, Woods DD, Leveson N (2006). Essential characteristics of resilience. Resilience engineering: concepts and precepts.

[46] Bernstein SL, Aronsky D, Duseja R, Epstein S, Handel D, Hwang U, McCarthy M, John McConnell K, Pines JM, Rathlev N, Schafermeyer R (2009). The effect of emergency department crowding on clinically oriented outcomes. Acad Emerg Med.

[47] Higginson I (2012). Emergency department crowding. Emerg Med J.

[48] King’s Fund. What’s going on in A&E? The key questions answered. http://www.kingsfund.org.uk/projects/urgent-emergency-care/urgent-and-emergency-care-mythbusters. Accessed 26 May 2016.

[49] Taylor DM, Wolfe R, Cameron PA (2002). Complaints from emergency department patients largely result from treatment and communication problems. Emerg Med.

[50] Oliver D. Doddery but a little dear? A defence of improving care [online]. 2010. Available at: http://www.battleofideas.org.uk/index.php/2010/battles/5401. Accessed 6 Oct 2016.

[51] Tadd W, Hillamn A, Calman S, Calman M, Bayer T, Read S (2011). Dignity in practice: an exploration of the care of older adults in acute NHS trusts.

[52] Tsilimingras D, Rosen AK, Berlowitz DR (2003). Patient safety in geriatrics: a call for action. J Gerontol A Biol Sci Med Sci.

[53] Maben J, Adams M, Peccei R, Murrells T, Robert G (2012). ‘Poppets and parcels’: the links between staff experience of work and acutely ill older peoples’ experience of hospital care. Int J Older People Nurs.

[54] Francis R (2013). Independent inquiry into care provided by Mid Staffordshire NHS Foundation Trust January 2005–March 2009.

[55] Strauss A, Corbin J, Denzin NK, Lincoln YS (1994). Grounded theory methodology. Handbook of qualitative research.

[56] Anderson R (2008). New MRC, guidance on evaluating complex interventions. BMJ.

[57] Mays N, Pope C (1995). Qualitative research: observational methods in health care settings. BMJ.

[58] Waterson P (2009). A critical review of the systems approach within patient safety research. Ergonomics.

[59] Tashakkori A, Teddlie C (2010). Sage handbook of mixed methods in social and behavioural research.

[60] Reeves S, Kuper A, Hodges BD (2008). Qualitative research methodologies: ethnography. BMJ.

[61] The Centre for Applied Resilience in Healthcare (CARe) website. www.resiliencecentre.org.uk. Accessed 28 Sept 2015.

